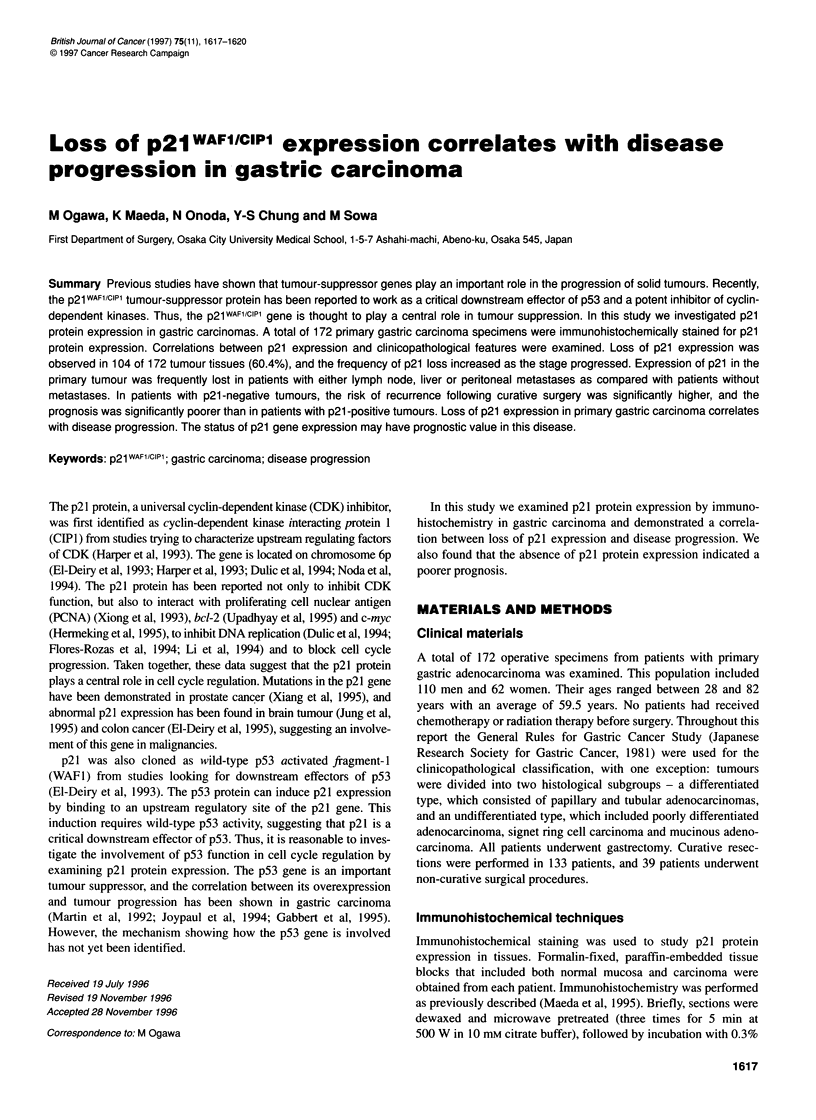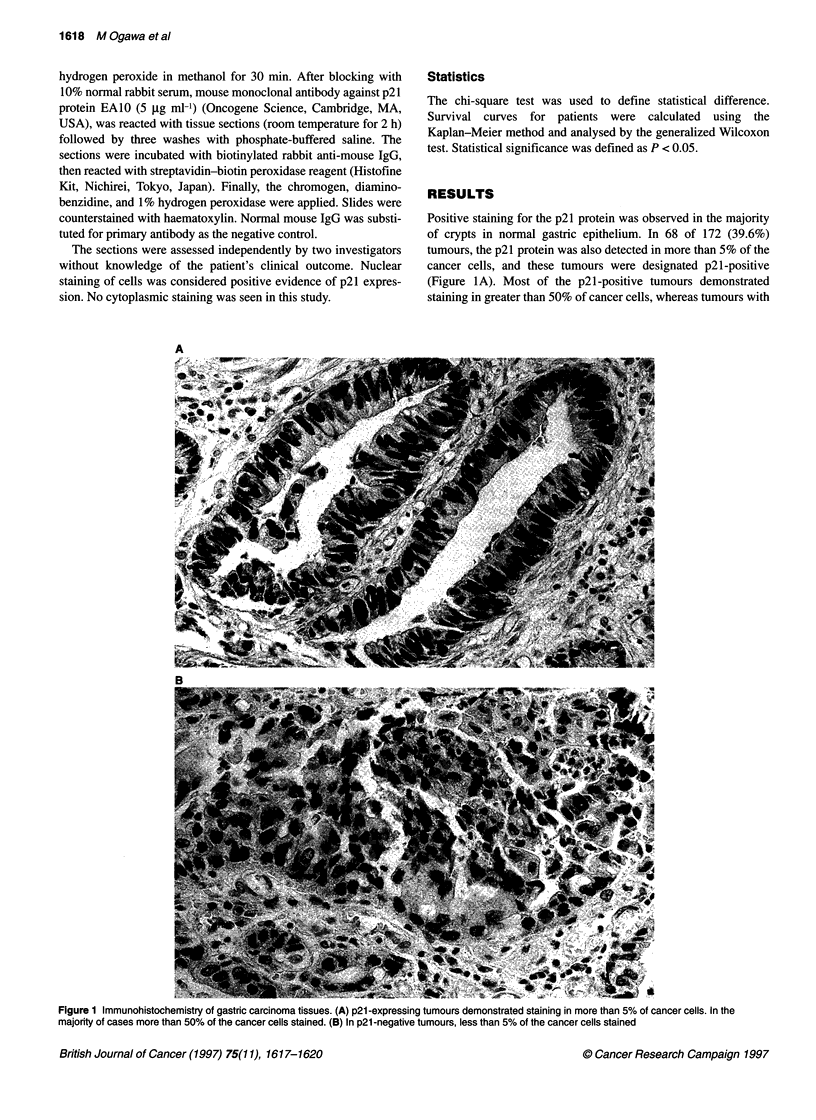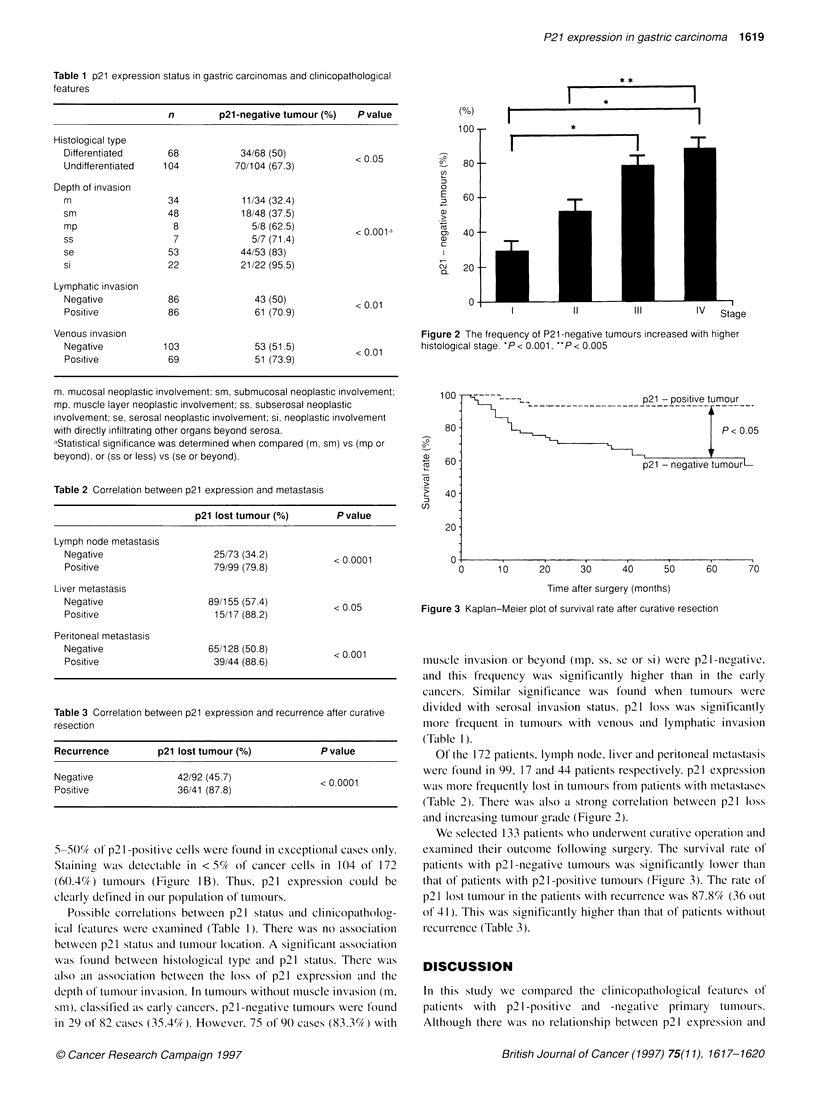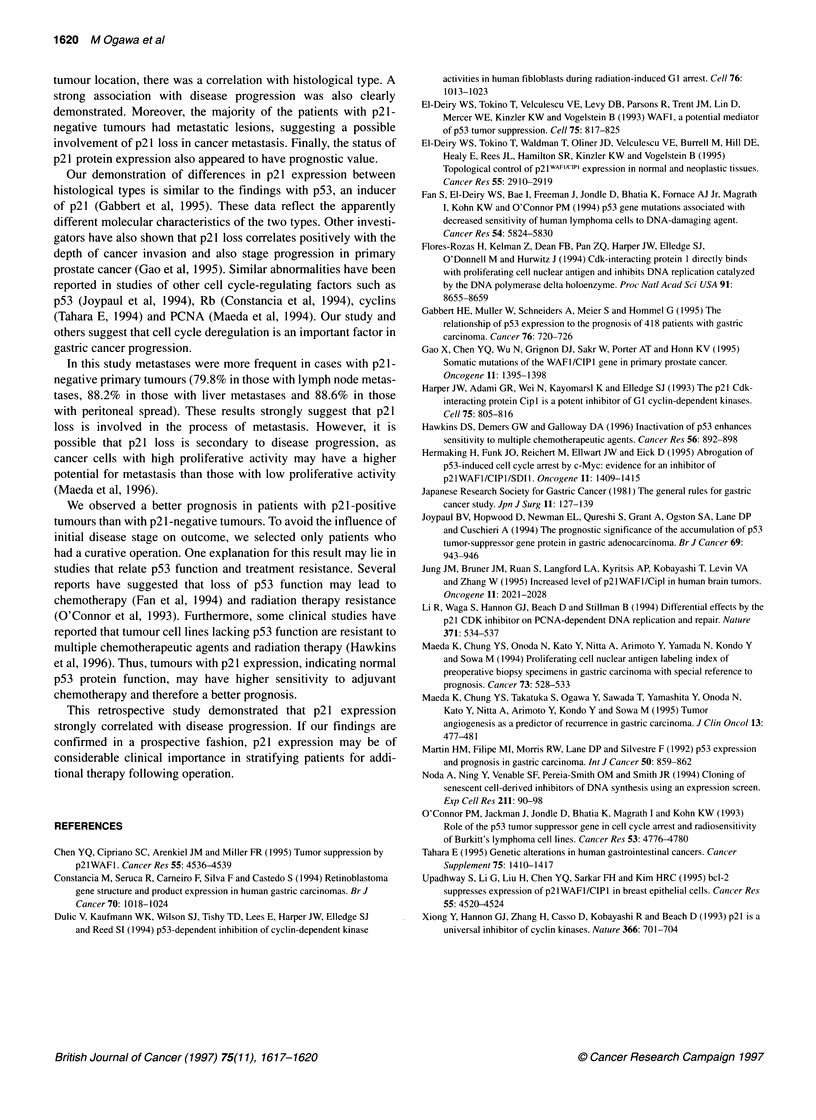# Loss of p21WAF1/CIP1 expression correlates with disease progression in gastric carcinoma.

**DOI:** 10.1038/bjc.1997.276

**Published:** 1997

**Authors:** M. Ogawa, K. Maeda, N. Onoda, Y. S. Chung, M. Sowa

**Affiliations:** First Department of Surgery, Osaka City University Medical School, Abeno-ku, Japan.

## Abstract

**Images:**


British Journal of Cancer (1997) 75(11), 1617–1620
© 1997 Cancer Research Campaign

### Loss of p21$^{WAF1/CIP1}$ expression correlates with disease progression in gastric carcinoma

**M Ogawa, K Maeda, N Onoda, Y-S Chung and M Sowa**

First Department of Surgery, Osaka City University Medical School, 1-5-7 Ashahi-machi, Abeno-ku, Osaka 545, Japan

**Summary** Previous studies have shown that tumour-suppressor genes play an important role in the progression of solid tumours. Recently, the p21$^{WAF1/CIP1}$ tumour-suppressor protein has been reported to work as a critical downstream effector of p53 and a potent inhibitor of cyclin-dependent kinases. Thus, the p21$^{WAF1/CIP1}$ gene is thought to play a central role in tumour suppression. In this study we investigated p21 protein expression in gastric carcinomas. A total of 172 primary gastric carcinoma specimens were immunohistochemically stained for p21 protein expression. Correlations between p21 expression and clinicopathological features were examined. Loss of p21 expression was observed in 104 of 172 tumour tissues (60.4%), and the frequency of p21 loss increased as the stage progressed. Expression of p21 in the primary tumour was frequently lost in patients with either lymph node, liver or peritoneal metastases as compared with patients without metastases. In patients with p21-negative tumours, the risk of recurrence following curative surgery was significantly higher, and the prognosis was significantly poorer than in patients with p21-positive tumours. Loss of p21 expression in primary gastric carcinoma correlates with disease progression. The status of p21 gene expression may have prognostic value in this disease.

**Keywords**: p21$^{WAF1/CIP1}$; gastric carcinoma; disease progression

The p21 protein, a universal cyclin-dependent kinase (CDK) inhibitor, was first identified as *c*yclin-dependent kinase *i*nteracting *p*rotein 1 (CIP1) from studies trying to characterize upstream regulating factors of CDK (Harper et al, 1993). The gene is located on chromosome 6p (El-Deiry et al, 1993; Harper et al, 1993; Dulic et al, 1994; Noda et al, 1994). The p21 protein has been reported not only to inhibit CDK function, but also to interact with proliferating cell nuclear antigen (PCNA) (Xiong et al, 1993), *bcl-2* (Upadhyay et al, 1995) and c-*myc* (Hermeking et al, 1995), to inhibit DNA replication (Dulic et al, 1994; Flores-Rozas et al, 1994; Li et al, 1994) and to block cell cycle progression. Taken together, these data suggest that the p21 protein plays a central role in cell cycle regulation. Mutations in the p21 gene have been demonstrated in prostate cancer (Xiang et al, 1995), and abnormal p21 expression has been found in brain tumour (Jung et al, 1995) and colon cancer (El-Deiry et al, 1995), suggesting an involvement of this gene in malignancies.

p21 was also cloned as *w*ild-type p53 *a*ctivated *f*ragment-1 (WAF1) from studies looking for downstream effectors of p53 (El-Deiry et al, 1993). The p53 protein can induce p21 expression by binding to an upstream regulatory site of the p21 gene. This induction requires wild-type p53 activity, suggesting that p21 is a critical downstream effector of p53. Thus, it is reasonable to investigate the involvement of p53 function in cell cycle regulation by examining p21 protein expression. The p53 gene is an important tumour suppressor, and the correlation between its overexpression and tumour progression has been shown in gastric carcinoma (Martin et al, 1992; Joypaul et al, 1994; Gabbert et al, 1995). However, the mechanism showing how the p53 gene is involved has not yet been identified.

*Received 19 July 1996*
*Revised 19 November 1996*
*Accepted 28 November 1996*

*Correspondence to:* M Ogawa

In this study we examined p21 protein expression by immunohistochemistry in gastric carcinoma and demonstrated a correlation between loss of p21 expression and disease progression. We also found that the absence of p21 protein expression indicated a poorer prognosis.

### MATERIALS AND METHODS

### Clinical materials

A total of 172 operative specimens from patients with primary gastric adenocarcinoma was examined. This population included 110 men and 62 women. Their ages ranged between 28 and 82 years with an average of 59.5 years. No patients had received chemotherapy or radiation therapy before surgery. Throughout this report the General Rules for Gastric Cancer Study (Japanese Research Society for Gastric Cancer, 1981) were used for the clinicopathological classification, with one exception: tumours were divided into two histological subgroups – a differentiated type, which consisted of papillary and tubular adenocarcinomas, and an undifferentiated type, which included poorly differentiated adenocarcinoma, signet ring cell carcinoma and mucinous adenocarcinoma. All patients underwent gastrectomy. Curative resections were performed in 133 patients, and 39 patients underwent non-curative surgical procedures.

### Immunohistochemical techniques

Immunohistochemical staining was used to study p21 protein expression in tissues. Formalin-fixed, paraffin-embedded tissue blocks that included both normal mucosa and carcinoma were obtained from each patient. Immunohistochemistry was performed as previously described (Maeda et al, 1995). Briefly, sections were dewaxed and microwave pretreated (three times for 5 min at 500 W in 10 mM citrate buffer), followed by incubation with 0.3%

hydrogen peroxide in methanol for 30 min. After blocking with 10% normal rabbit serum, mouse monoclonal antibody against p21 protein EA10 (5 μg $ml^{-1}$) (Oncogene Science, Cambridge, MA, USA), was reacted with tissue sections (room temperature for 2 h) followed by three washes with phosphate-buffered saline. The sections were incubated with biotinylated rabbit anti-mouse IgG, then reacted with streptavidin–biotin peroxidase reagent (Histofine Kit, Nichirei, Tokyo, Japan). Finally, the chromogen, diaminobenzidine, and 1% hydrogen peroxidase were applied. Slides were counterstained with haematoxylin. Normal mouse IgG was substituted for primary antibody as the negative control.

The sections were assessed independently by two investigators without knowledge of the patient's clinical outcome. Nuclear staining of cells was considered positive evidence of p21 expression. No cytoplasmic staining was seen in this study.

### Statistics

The chi-square test was used to define statistical difference. Survival curves for patients were calculated using the Kaplan–Meier method and analysed by the generalized Wilcoxon test. Statistical significance was defined as $P < 0.05$.

### RESULTS

Positive staining for the p21 protein was observed in the majority of crypts in normal gastric epithelium. In 68 of 172 (39.6%) tumours, the p21 protein was also detected in more than 5% of the cancer cells, and these tumours were designated p21-positive (Figure 1A). Most of the p21-positive tumours demonstrated staining in greater than 50% of cancer cells, whereas tumours with

A

B

**Figure 1** Immunohistochemistry of gastric carcinoma tissues. (**A**) p21-expressing tumours demonstrated staining in more than 5% of cancer cells. In the majority of cases more than 50% of the cancer cells stained. (**B**) In p21-negative tumours, less than 5% of the cancer cells stained

*© Cancer Research Campaign 1997*

**Table 1** p21 expression status in gastric carcinomas and clinicopathological features

| | *n* | p21-negative tumour (%) | *P* value |
|---|---|---|---|
| Histological type | | | |
| Differentiated | 68 | 34/68 (50) | < 0.05 |
| Undifferentiated | 104 | 70/104 (67.3) | |
| Depth of invasion | | | |
| m | 34 | 11/34 (32.4) | < 0.001[a] |
| sm | 48 | 18/48 (37.5) | |
| mp | 8 | 5/8 (62.5) | |
| ss | 7 | 5/7 (71.4) | |
| se | 53 | 44/53 (83) | |
| si | 22 | 21/22 (95.5) | |
| Lymphatic invasion | | | |
| Negative | 86 | 43 (50) | < 0.01 |
| Positive | 86 | 61 (70.9) | |
| Venous invasion | | | |
| Negative | 103 | 53 (51.5) | < 0.01 |
| Positive | 69 | 51 (73.9) | |

m, mucosal neoplastic involvement; sm, submucosal neoplastic involvement; mp, muscle layer neoplastic involvement; ss, subserosal neoplastic involvement; se, serosal neoplastic involvement; si, neoplastic involvement with directly infiltrating other organs beyond serosa.
[a]Statistical significance was determined when compared (m, sm) vs (mp or beyond), or (ss or less) vs (se or beyond).

**Table 2** Correlation between p21 expression and metastasis

| | p21 lost tumour (%) | *P* value |
|---|---|---|
| Lymph node metastasis | | |
| Negative | 25/73 (34.2) | < 0.0001 |
| Positive | 79/99 (79.8) | |
| Liver metastasis | | |
| Negative | 89/155 (57.4) | < 0.05 |
| Positive | 15/17 (88.2) | |
| Peritoneal metastasis | | |
| Negative | 65/128 (50.8) | < 0.001 |
| Positive | 39/44 (88.6) | |

**Table 3** Correlation between p21 expression and recurrence after curative resection

| Recurrence | p21 lost tumour (%) | *P* value |
|---|---|---|
| Negative | 42/92 (45.7) | < 0.0001 |
| Positive | 36/41 (87.8) | |

5–50% of p21-positive cells were found in exceptional cases only. Staining was detectable in < 5% of cancer cells in 104 of 172 (60.4%) tumours (Figure 1B). Thus, p21 expression could be clearly defined in our population of tumours.

Possible correlations between p21 status and clinicopathological features were examined (Table 1). There was no association between p21 status and tumour location. A significant association was found between histological type and p21 status. There was also an association between the loss of p21 expression and the depth of tumour invasion. In tumours without muscle invasion (m, sm), classified as early cancers, p21-negative tumours were found in 29 of 82 cases (35.4%). However, 75 of 90 cases (83.3%) with muscle invasion or beyond (mp, ss, se or si) were p21-negative, and this frequency was significantly higher than in the early cancers. Similar significance was found when tumours were divided with serosal invasion status. p21 loss was significantly more frequent in tumours with venous and lymphatic invasion (Table 1).

**Figure 2** The frequency of P21-negative tumours increased with higher histological stage. $^{*}P < 0.001$, $^{**}P < 0.005$

**Figure 3** Kaplan–Meier plot of survival rate after curative resection

Of the 172 patients, lymph node, liver and peritoneal metastasis were found in 99, 17 and 44 patients respectively. p21 expression was more frequently lost in tumours from patients with metastases (Table 2). There was also a strong correlation between p21 loss and increasing tumour grade (Figure 2).

We selected 133 patients who underwent curative operation and examined their outcome following surgery. The survival rate of patients with p21-negative tumours was significantly lower than that of patients with p21-positive tumours (Figure 3). The rate of p21 lost tumour in the patients with recurrence was 87.8% (36 out of 41). This was significantly higher than that of patients without recurrence (Table 3).

### DISCUSSION

In this study we compared the clinicopathological features of patients with p21-positive and -negative primary tumours. Although there was no relationship between p21 expression and

© Cancer Research Campaign 1997

tumour location, there was a correlation with histological type. A strong association with disease progression was also clearly demonstrated. Moreover, the majority of the patients with p21-negative tumours had metastatic lesions, suggesting a possible involvement of p21 loss in cancer metastasis. Finally, the status of p21 protein expression also appeared to have prognostic value.

Our demonstration of differences in p21 expression between histological types is similar to the findings with p53, an inducer of p21 (Gabbert et al, 1995). These data reflect the apparently different molecular characteristics of the two types. Other investigators have also shown that p21 loss correlates positively with the depth of cancer invasion and also stage progression in primary prostate cancer (Gao et al, 1995). Similar abnormalities have been reported in studies of other cell cycle-regulating factors such as p53 (Joypaul et al, 1994), Rb (Constancia et al, 1994), cyclins (Tahara E, 1994) and PCNA (Maeda et al, 1994). Our study and others suggest that cell cycle deregulation is an important factor in gastric cancer progression.

In this study metastases were more frequent in cases with p21-negative primary tumours (79.8% in those with lymph node metastases, 88.2% in those with liver metastases and 88.6% in those with peritoneal spread). These results strongly suggest that p21 loss is involved in the process of metastasis. However, it is possible that p21 loss is secondary to disease progression, as cancer cells with high proliferative activity may have a higher potential for metastasis than those with low proliferative activity (Maeda et al, 1996).

We observed a better prognosis in patients with p21-positive tumours than with p21-negative tumours. To avoid the influence of initial disease stage on outcome, we selected only patients who had a curative operation. One explanation for this result may lie in studies that relate p53 function and treatment resistance. Several reports have suggested that loss of p53 function may lead to chemotherapy (Fan et al, 1994) and radiation therapy resistance (O'Connor et al, 1993). Furthermore, some clinical studies have reported that tumour cell lines lacking p53 function are resistant to multiple chemotherapeutic agents and radiation therapy (Hawkins et al, 1996). Thus, tumours with p21 expression, indicating normal p53 protein function, may have higher sensitivity to adjuvant chemotherapy and therefore a better prognosis.

This retrospective study demonstrated that p21 expression strongly correlated with disease progression. If our findings are confirmed in a prospective fashion, p21 expression may be of considerable clinical importance in stratifying patients for additional therapy following operation.

### REFERENCES

Chen YQ, Cipriano SC, Arenkiel JM and Miller FR (1995) Tumor suppression by p21WAF1. *Cancer Res* **55**: 4536–4539

Constancia M, Seruca R, Carneiro F, Silva F and Castedo S (1994) Retinoblastoma gene structure and product expression in human gastric carcinomas. *Br J Cancer* **70**: 1018–1024

Dulic V, Kaufmann WK, Wilson SJ, Tishy TD, Lees E, Harper JW, Elledge SJ and Reed SI (1994) p53-dependent inhibition of cyclin-dependent kinase activities in human fibloblasts during radiation-induced G1 arrest. *Cell* **76**: 1013–1023

El-Deiry WS, Tokino T, Velculescu VE, Levy DB, Parsons R, Trent JM, Lin D, Mercer WE, Kinzler KW and Vogelstein B (1993) WAF1, a potential mediator of p53 tumor suppression. *Cell* **75**: 817–825

El-Deiry WS, Tokino T, Waldman T, Oliner JD, Velculescu VE, Burrell M, Hill DE, Healy E, Rees JL, Hamilton SR, Kinzler KW and Vogelstein B (1995) Topological control of $p21^{WAF1/CIP1}$ expression in normal and neoplastic tissues. *Cancer Res* **55**: 2910–2919

Fan S, El-Deiry WS, Bae I, Freeman J, Jondle D, Bhatia K, Fornace AJ Jr, Magrath I, Kohn KW and O'Connor PM (1994) p53 gene mutations associated with decreased sensitivity of human lymphoma cells to DNA-damaging agent. *Cancer Res* **54**: 5824–5830

Flores-Rozas H, Kelman Z, Dean FB, Pan ZQ, Harper JW, Elledge SJ, O'Donnell M and Hurwitz J (1994) Cdk-interacting protein 1 directly binds with proliferating cell nuclear antigen and inhibits DNA replication catalyzed by the DNA polymerase delta holoenzyme. *Proc Natl Acad Sci USA* **91**: 8655–8659

Gabbert HE, Muller W, Schneiders A, Meier S and Hommel G (1995) The relationship of p53 expression to the prognosis of 418 patients with gastric carcinoma. *Cancer* **76**: 720–726

Gao X, Chen YQ, Wu N, Grignon DJ, Sakr W, Porter AT and Honn KV (1995) Somatic mutations of the WAF1/CIP1 gene in primary prostate cancer. *Oncogene* **11**: 1395–1398

Harper JW, Adami GR, Wei N, Kayomarsl K and Elledge SJ (1993) The p21 Cdk-interacting protein Cip1 is a potent inhibitor of G1 cyclin-dependent kinases. *Cell* **75**: 805–816

Hawkins DS, Demers GW and Galloway DA (1996) Inactivation of p53 enhances sensitivity to multiple chemotherapeutic agents. *Cancer Res* **56**: 892–898

Hermaking H, Funk JO, Reichert M, Ellwart JW and Eick D (1995) Abrogation of p53-induced cell cycle arrest by c-Myc: evidence for an inhibitor of p21WAF1/CIP1/SDI1. *Oncogene* **11**: 1409–1415

Japanese Research Society for Gastric Cancer (1981) The general rules for gastric cancer study. *Jpn J Surg* **11**: 127–139

Joypaul BV, Hopwood D, Newman EL, Qureshi S, Grant A, Ogston SA, Lane DP and Cuschieri A (1994) The prognostic significance of the accumulation of p53 tumor-suppressor gene protein in gastric adenocarcinoma. *Br J Cancer* **69**: 943–946

Jung JM, Bruner JM, Ruan S, Langford LA, Kyritsis AP, Kobayashi T, Levin VA and Zhang W (1995) Increased level of p21WAF1/Cipl in human brain tumors. *Oncogene* **11**: 2021–2028

Li R, Waga S, Hannon GJ, Beach D and Stillman B (1994) Differential effects by the p21 CDK inhibitor on PCNA-dependent DNA replication and repair. *Nature* **371**: 534–537

Maeda K, Chung YS, Onoda N, Kato Y, Nitta A, Arimoto Y, Yamada N, Kondo Y and Sowa M (1994) Proliferating cell nuclear antigen labeling index of preoperative biopsy specimens in gastric carcinoma with special reference to prognosis. *Cancer* **73**: 528–533

Maeda K, Chung YS, Takatuka S, Ogawa Y, Sawada T, Yamashita Y, Onoda N, Kato Y, Nitta A, Arimoto Y, Kondo Y and Sowa M (1995) Tumor angiogenesis as a predictor of recurrence in gastric carcinoma. *J Clin Oncol* **13**: 477–481

Martin HM, Filipe MI, Morris RW, Lane DP and Silvestre F (1992) p53 expression and prognosis in gastric carcinoma. *Int J Cancer* **50**: 859–862

Noda A, Ning Y, Venable SF, Pereia-Smith OM and Smith JR (1994) Cloning of senescent cell-derived inhibitors of DNA synthesis using an expression screen. *Exp Cell Res* **211**: 90–98

O'Connor PM, Jackman J, Jondle D, Bhatia K, Magrath I and Kohn KW (1993) Role of the p53 tumor suppressor gene in cell cycle arrest and radiosensitivity of Burkitt's lymphoma cell lines. *Cancer Res* **53**: 4776–4780

Tahara E (1995) Genetic alterations in human gastrointestinal cancers. *Cancer Supplement* **75**: 1410–1417

Upadhway S, Li G, Liu H, Chen YQ, Sarkar FH and Kim HRC (1995) bcl-2 suppresses expression of p21WAF1/CIP1 in breast epithelial cells. *Cancer Res* **55**: 4520–4524

Xiong Y, Hannon GJ, Zhang H, Casso D, Kobayashi R and Beach D (1993) p21 is a universal inhibitor of cyclin kinases. *Nature* **366**: 701–704

© Cancer Research Campaign 1997